# American Academy of Medicine

**Published:** 1883-09-20

**Authors:** Richard J. Dunglison

**Affiliations:** Secretary


### *AMERICAN ACADEMY OF MEDICINE.*

The Annual Meeting of the Academy will be held at the New York Academy of Medicine, 12 W. 31st Street, New York, on Tuesday, October 9th, (three o'clock P. M.), and Wednesday, October 10th 1883.

RICHARD J. DUNGLISON, *Secretary.*

